# Palisaded Neutrophilic and Granulomatous Dermatitis in a Patient With Churg-Strauss Syndrome: A Case Report and Literature Review

**DOI:** 10.7759/cureus.30085

**Published:** 2022-10-08

**Authors:** Yaser Mansoor Almutawa, Walaa Alherz, Mayyasa Osama Alali, Aalaa Mubarak, Ameen Al Awadhi

**Affiliations:** 1 Department of Dermatology and Venerology, Salmaniya Medical Complex, Manama, BHR; 2 Department of Pathology, Salmaniya Medical Complex, Manama, BHR

**Keywords:** extravascular necrotizing granuloma and hypereosinophilia, asthma, eosinophilic granulomatosis with polyangiitis (egpa), churg-strauss syndrome (css), palisaded neutrophilic and granulomatous dermatitis (pngd)

## Abstract

Palisaded neutrophilic and granulomatous dermatitis (PNGD) is a rare reaction pattern that is frequently linked to several systemic diseases, including autoimmune disease, inflammatory bowel disease, and vasculitis. Churg-Strauss syndrome (CSS) is an uncommon systemic condition that occurs exclusively in patients with asthma or a history of atopy. It is characterized by extravascular necrotizing granuloma and hypereosinophilia. This case report describes an illustrative case of a 61-year-old Bahraini female who had been diagnosed with CSS and presented with PNGD. The PNGD appeared a few weeks after her oral corticosteroid medication was discontinued. The present case report aims to assist in accurately diagnosing PNGD, as rarely manifested in this case, thus aiding clinicians in improving patient care.

## Introduction

Skin manifestation will be present in about 50% of Churg-Strauss syndrome (CSS) cases (in some literature, 40-81%) [1]. These manifestations can be hemorrhagic lesions [2], including purpura, petechiae [3], ecchymosis, and hemorrhagic bullae, or they may present as dermal or subcutaneous papules and nodules. The most common location for these manifestations is the extensor surface of both lower extremities [3]. The upper extremities and scalp are common locations as well [2]. Other less observed manifestations include urticaria, hypoesthesia [4], erythematous macules, and livedo reticularis [1].

All reviewed literature cases mainly described necrotizing palisaded granulomas and leukocytoclastic vasculitis [3]. Dermal infiltration with neutrophils, extravasated red blood cells, and numerous eosinophils are also common findings. Some cases demonstrate marked eosinophilic inflammatory infiltrates mainly around the dermis's nerve fibers, which gives a mononeuropathy picture [4,5]. Few cases will reveal strands of degenerative collagen fibers along with increased dermal mucin. Palisaded neutrophilic and granulomatous dermatitis (PNGD) is a benign dermatosis with specific histopathological characteristics often detected in patients with systemic conditions. PNGD is seen in people of all ages, and women are affected more frequently due to the systemic disorders linked to PNGD [6,7]; however, reports among children are rare [8].

CSS is an eosinophilic infiltration of tissues that causes disorders involving inflammation of the blood vessel walls [9]. The specimens usually received in the lab and diagnosed histologically as CSS are kidney core biopsies, lung biopsies, and skin biopsies. All these biopsies will show more or less the same histological findings. Alongside the above-mentioned histopathological features of CSS seen under the microscope, the lung will also show prominent eosinophilic infiltrate/eosinophilic abscess, while the kidney will demonstrate crescentic glomerulonephritis [10]. To rule out other forms of vasculitis, it is usually necessary to correlate clinical and serological findings. It is important to note that this study reports a case of PNGD in a female patient with CSS.

## Case presentation

A 61-year-old Bahraini female was diagnosed with asthma in 2000 and chest symptoms that were being worked up for a systemic disease with Eosinophilic Granulomatosis with Polyangiitis (known as Churg-Strauss Syndrome), in which she was diagnosed previously. She was referred to the Dermatology department for consultation during her hospital admission for multiple papules around the elbow joints that had lasted for one month. They appeared a few weeks after she discontinued her oral corticosteroid medication; it was pruritic, erythematous, and painful. Multiple red papules were present and mainly localized around the elbow joints. The pigment of the papules evolves from red to black as they develop dryness and crust before finally resolving. No other associated symptoms were noted. The patient noted that her quality of life was affected, as it painfully prevented her from resuming daily activities, but she would find relief with oral corticosteroids.

The skin rash first appeared in 2003 at the time of diagnosis. The areas affected were the same as those now, but they were more extensive, involving palms, soles, shins, and buttocks. They had the same characteristics. After seeking medical advice from Jordan, the patient started an oral corticosteroid. She was admitted multiple times for nasal polyposis and asthma. Her current admission was for bilateral lower limb swelling for investigation.

**Figure 1:**
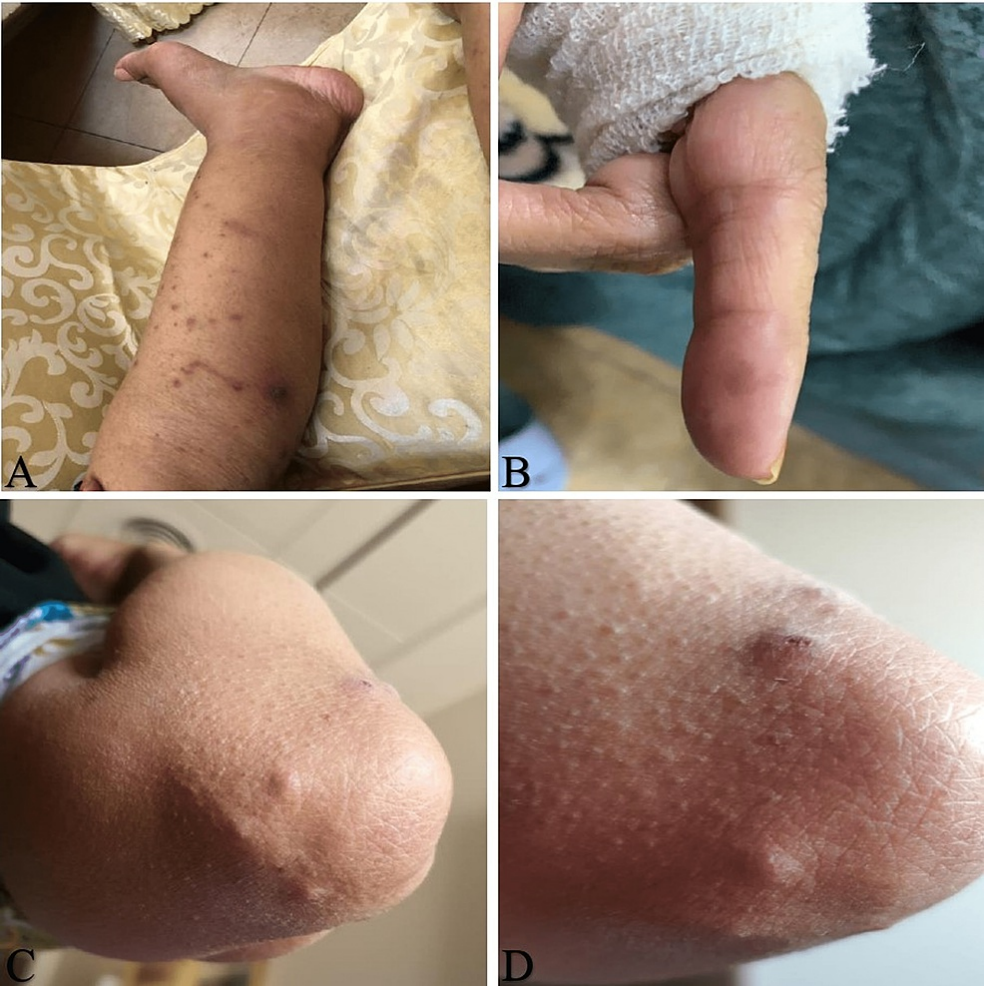
Multiple tender erythematous papules A. Skin lesion of the lateral lower extremity – shows edema and widespread non-palpable purpura with central single lesion necrosis. B. Single subcutaneous nodule of the index finger. C. & D. Raised lesion of the elbow – multiple tender erythematous, purpuric nodules with overlying erosions and crusts on the elbow.

On clinical examination, multiple tender erythematous crusted papules were seen around the elbow joints (Figure 1). The abdomen was soft, with no organomegaly noted, despite the previous scarring caused by the relapse of the disease in the areas mentioned above. She had bilateral lower limb edema with mild tenderness.

**Figure 2:**
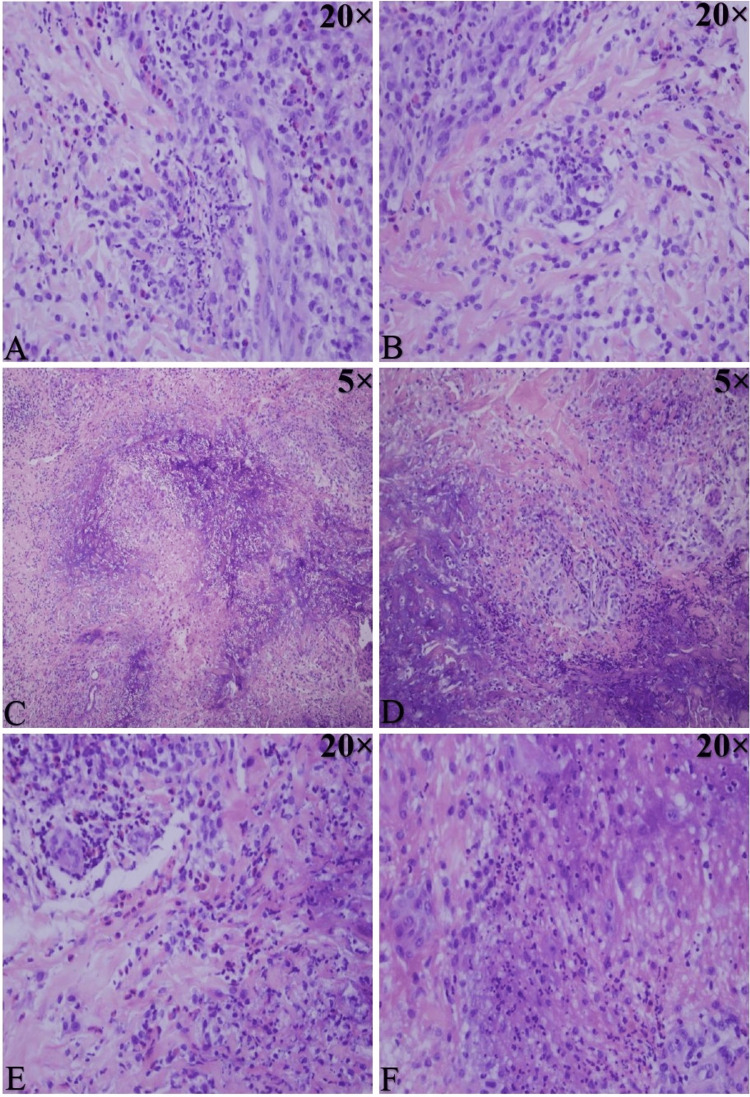
Histopathology of a punch elbow biopsy show heavy interstitial granulomatous dermatitis and focal palisading around foci of the neutrophilic collection and leukocytoclasia Focal areas might lead to degenerating collagen and increased dermal mucin. A. & B. show features of vasculitis. C. & D. Palisaded granuloma. E. Inflammation - rich in eosinophils. F. Karyorrhectic debris.

The laboratory tests showed positive eosinophilia and the IgE inhalation panel. In contrast, radiological examination showed apical bronchiectasis alterations. The patient was again started on oral corticosteroid, fusidic acid, betamethasone, and valerate cream for the active papules. Before the initiation of treatment, two punch biopsies were obtained from the patient elbows and sent for histopathology investigations (Figure 2). Special stains for mycobacteria and fungal profiles were negative. Based on the above-mentioned clinicopathological investigations, a diagnosis of PNGD was made.

## Discussion

Here, we described a case of PNGD in a female with Churg-Strauss Syndrome. We believe that a physician should consider the diagnosis of PNGD in patients with CSS that present with cutaneous eruptions accompanied by marked eosinophilia and high IgE levels. The origin and cause of PNGD in CSS patients for the remarkably higher eosinophil levels throughout the dermis are poorly understood.

PNGD is an uncommon disease linked to specific clinicopathological characteristics. PNGD was identified as allergic granulomatosis in 1951 [11]. Later on, in 1994 Chu, et al. recognized it as PNGD [12].

The pathogenesis of PNGD is poorly understood and remains unknown. Some reports have suggested immune complex deposition [12]. Recently, Deen et al. have indicated that the granulomas may be a nonspecific immunological response likely associated with the underlying disorder [13]. Clinically, PNGD presents erythematous papules with crusting and symmetrical distribution of tender erythematous to violaceous papules [8]. Histologic findings usually vary based on the stage of the disease. Palisading granulomas are more frequently encountered in mature lesions of PNGD. Lymphohistiocytic cells with infiltration of neutrophils in the derm were identified in early-stage biopsies, while late-stage biopsies include scattered leukocytes, granulomas, fibrin, neutrophil debris, and ultimately fibrosis [14]. One patient may present distinctive biopsies under the microscope [15].

The clinical differential diagnosis includes leukocytoclastic vasculitis, urticaria, interstitial granulomatous dermatitis, and granuloma annulare [16,17]. The pathologic characteristics enable the diseases to be differentiated. Pathologic differential diagnoses include bowel-associated dermatosis-arthritis syndrome, rheumatoid nodules, Sweet’s syndrome pyoderma gangrenosum, and leukocytoclastic vasculitis [18,19].

PNGD is a benign condition, and its management depends on treating the underlying disease. Diverse therapeutic options are available, including colchicine, systemic corticosteroids, dapsone, hydroxychloroquine, and cyclosporine [20].

## Conclusions

PNGD is a rare cutaneous disease, which is usually linked to systemic medication or an underlying condition. It is well documented that PNGD occurs frequently in Churg-Strauss Syndrome. Hence, identifying PNGD in a patient with CSS has clinical significance because it is a skin marker of systemic disorder. Further studies are needed to gain insight into the pathogenesis of PNGD among patients with CSS.
